# Interactions of extracts from selected chewing stick sources with *Aggregatibacter actinomycetemcomitans*

**DOI:** 10.1186/1756-0500-5-203

**Published:** 2012-07-10

**Authors:** Francis Kwamin, Rolf Gref, Dorte Haubek, Anders Johansson

**Affiliations:** 1 Dental School University of Ghana, Accra, Ghana; 2 Swedish University of Agricultural Sciences, Faculty of Forestry, Umeå, Sweden; 3 Department of Dentistry, Aarhus University, Aarhus, Denmark; 4 Molecular Periodontology/Odontology, Umeå University, Umeå, Sweden; 5 Division of Molecular Periodontology, Department of Odontology, Faculty of Medicine, Umeå University, Umeå, Sweden

## Abstract

**Background:**

*Aggregatibacter actinomycetemcomitans* produces a leukotoxin that activates a pro-inflammatory death of human monocytes/macrophages. A specific clone of this bacterium (JP2) has a 530-base pair deletion in the leukotoxin promoter gene and significantly enhanced expression of leukotoxin. This specific clone of *A. actinomycetemcomitans* is common in some African populations and has a strong association with periodontal attachment loss in adolescents in these populations. Chewing sticks of plant origin are commonly used as oral hygiene tool in Africa, but their role as a therapeutic agent in periodontal disease is poorly investigated.

**Results:**

Ethanol extracts were made from 7 common plants used as chewing sticks in West-Africa. None of the tested extracts inhibited growth of *A. actinomycetemcomitans*. However, extracts from *Psidium guajava* (Guava) completely neutralized the cell death and pro-inflammatory response of human leukocytes induced by the leukotoxin. None of the six other tested chewing stick extracts showed this effect.

**Conclusions:**

The discovery that extracts from Guava efficiently neutralizes *A. actinomycetemcomitans* leukotoxicity might lead to novel therapeutic agents and strategies for prevention and treatment of aggressive forms of periodontitis induced by infections with the highly leukotoxic JP2 clone of this bacterium.

## Background

*Aggregatibacter actinomycetemcomitans* has been described as a member of the indigenous oral microbiota of humans, and it is involved in the pathology of periodontitis and various non-oral infections [1]. This bacterium selectively kills human leukocytes through expression of leukotoxin, a large pore-forming protein that belongs to the RTX family [2,3]. The specificity of the toxin is related to its prerequisite for a specific target-cell receptor, the lymphocyte function associated antigen 1 (LFA-1), which is solely expressed on leukocytes [4]. The leukotoxin causes death of different leukocyte populations in a variety of ways. It activates a rapid release of lysosomal enzymes and matrix metalloproteinases from neutrophils and causes apoptosis in lymphocytes [5-7]. In monocytes/macrophages, the toxin activates caspase-1, a cysteine proteinase, which causes a pro-inflammatory response by the activation and secretion of interleukin (IL)-1β and IL-18 [8-10]. These cellular and molecular activations are associated with the pathogenic mechanism of periodontitis, which results in loss of tooth-supporting tissues, bone and connective tissue [8,11]. A specific clone (JP2) of *A. actinomycetemcomitans* with enhanced leukotoxin expression is significantly associated with disease onset in infected individuals [12]. The JP2 clone is likely to have its origin in the Mediterranean part of Africa, but today it can be found occasionally also in South and North America and in Europe [13-15].

It has recently been shown that the occurrence of JP2 clone strains of *A. actinomycetemcomitans* in Ghanaian adolescents is high [16]. In this part of the world, the use of chewing sticks from different tree species has a long tradition as a tool for oral hygiene practices [17,18]. The sticks are chewed for mechanical removal of tooth plaque, but some plants used for this purpose might also have antimicrobial properties [19-22].

The aim of the present study was to evaluate if some of the commonly used chewing stick materials in Ghana have properties that could be used in therapeutic agents and for prevention strategies, and in treatment of infections with the highly leukotoxic clone (JP2) of *A. actinomycetemcomitans*.

## Results

### Antimicrobial screening

None of the seven tested chewing stick extracts showed any growth inhibitory effect on *A. actinomycetemcomitans* in either of the two *in vitro* test methods employed in the present study, growth on agar or growth in broth. Two different strains of this bacterium were used (D7SS and HK1519), in order to represent both JP2 and non-JP2 clonal types of *A. actinomycetemcomitans*. No inhibition zones were seen when 10 μl from each of the 7 concentrated stick extracts was added to agar plates inoculated with D7SS and HK1519. Also, no inhibitory effects on growth of these two strains were seen when ≥1% from each concentrated stick extracts were added and optical density was followed continuously during 48 h in the broth cultures.

### Neutralization of leukotoxin-induced cell death

**Figure 1:**
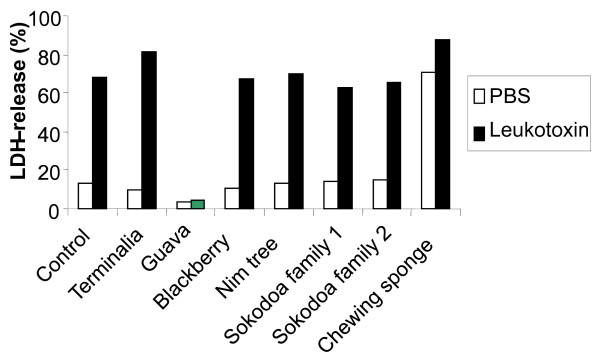
**Guava extracts block the leukotoxin-induced leukocyte lysis.** LDH-release from human mononuclear leukocytes exposed to *A. actinomycetemcomitans* leukotoxin (100 ng/ml) for 60 min with or without extracts (0.1%) from different chewing stick materials. Representative results are shown.

**Figure 2:**
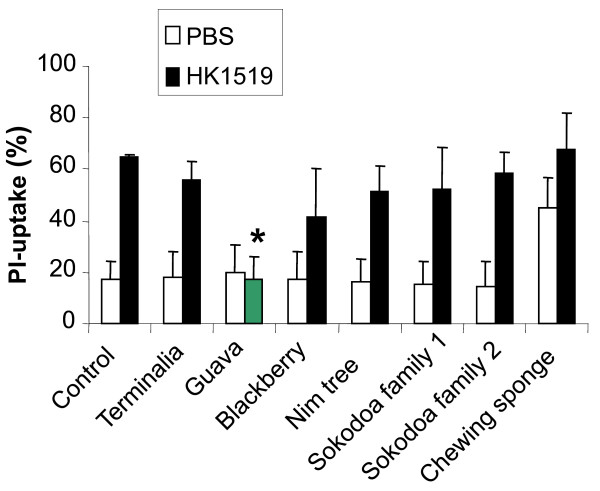
**Leukocytes survive exposure to highly leukotoxic*A. actinomycetemcomitans*in the presence of Guava.** Viability (propidium iodide-(PI) uptake) of human mononuclear leukocytes exposed to *A. actinomycetemcomitans* from the highly leukotoxic strain HK1519 in a ratio of 10 bacteria/leukocyte for 60 min in presence or absence of extracts (0.1%) from different chewing stick materials. The proportion of PI-stained dead cells was quantified by flow cytometry analyses. Mean ± SD of 3 experiments is shown and significant leukotoxin neutralization is indicated (*).

When the different chewing stick extracts were added to human mononuclear leukocytes that were exposed to *A. actinomycetemcomitans* leukotoxin, the induced LDH release was completely inhibited in samples containing 0.1% Guava twig extract (Figure 1). None of the other 6 extracts inhibited leukotoxicity at the concentration used (0.1%) in the present assay. The chewing sponge extract also caused LDH release in the absence of leukotoxin. When the mononuclear leukocytes were exposed to highly leukotoxic *A. actinomycetemcomitans*, cell death was examined with propidium iodide staining documented by flow cytometry. These analyses further indicated the leukotoxin-neutralizing capacity of the Guava twig extract, while none of the other tested extracts significantly inhibited the leukotoxicity (p = 0.006) (Figure 2). Chewing sponge alone also showed cytotoxic activity in this assay.

**Figure 3:**
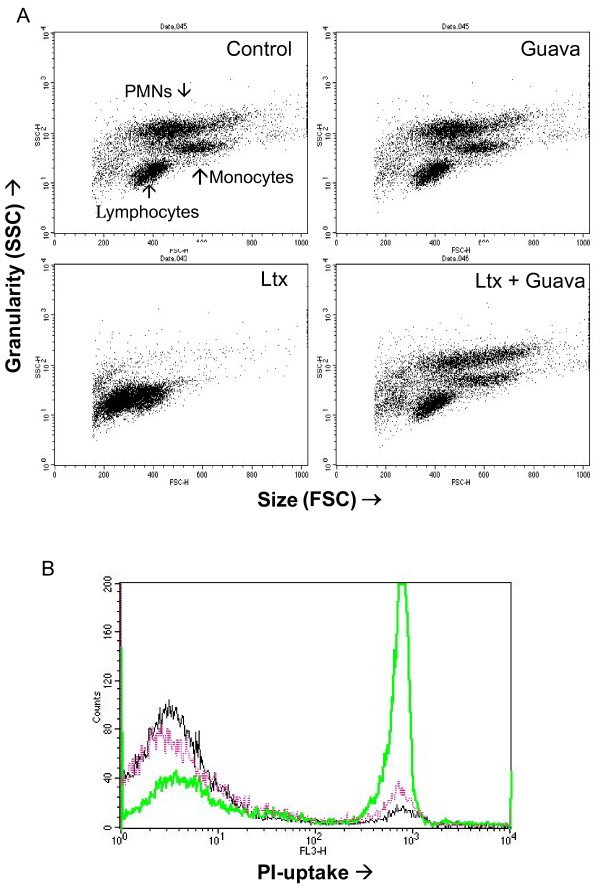
**Leukotoxin-induced morphological alterations in monocytes and neutrophils are abolished in the presence of Guava extract.** Human leukocytes exposed to *A. actinomycetemcomitans* leukotoxin (100 ng/ml) for 60 min with or without extracts (0.1%) from Guava twigs. Cell morphology of the different exposed leukocyte subsets (FSC/SSC) was documented by flow cytometry analyses (**A**). Viability (PI-uptake) in the exposed leukocyte populations was quantified by flow cytometry analyses (**B**). Black line represents control cells, green line leukotoxin-exposed cells and the violet line cells exposed to leukotoxin in the presence of Guava extract. Representative dot plots and histogram are shown.

**Figure 4:**
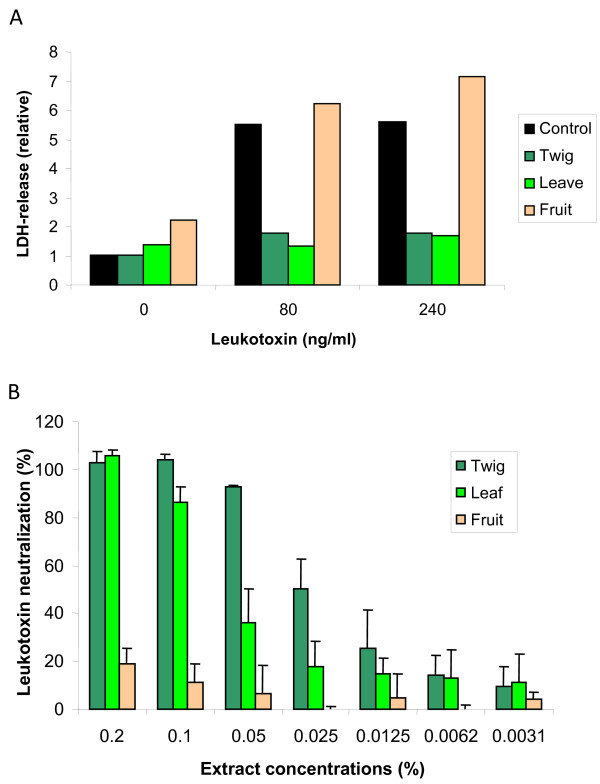
**The leukotoxin-neutralizing compounds of Guava are present in leaves and twigs, but not in the fruit.** (**A**) LDH-release from human leukocytes exposed to *A. actinomycetemcomitans leukotoxin* (0, 80, or 240 ng/ml) for 60 min in the presence of Guava extracts (0.1%) from twigs, leafs or fruits. Representative results of relative LDH release are shown. (**B**) Leukotoxin neutralizing capacity in different concentrations of Guava extracts (twigs, leafs, fruits) based on LDH-release from macrophages exposed to leukotoxin (80 ng/ml) for 60 min. Mean ± SD of 3 observations is shown.

Morphological analyses of leukotoxin-exposed human leukocytes by flow cytometry showed that polymorphonuclear leukocytes (PMNs) and monocytes were sensitive targets for the leukotoxin (Figure 3A). These morphological alterations induced by the leukotoxin were completely abolished in the presence of Guava extract (0.1%). Propidium iodide staining of the exposed leukocytes further indicated the protective effect of the Guava extract. In a first attempt to localize the presence of the responsible compounds in the plant, extracts from fruit and leaves were also examined. Extracts from leaves (0.1%) and twigs (0.1%) showed similar leukotoxin-neutralizing capacity, while the fruit extract (0.1%) completely lacked such capacity (Figure 4). A dose–response experiment showed that the extract from the twigs had a higher capacity to neutralize leukotoxin than the extracts from the leaves (Figure 4B).

In order to validate the stability of the results, extracts from Guava plants were made from materials collected both in the Greater Accra and the Volta regions of Ghana. The region where the plant was picked did not interfere with the ability of the extract to neutralize *A. actinomycetemcomitans* leukotoxin.

### Neutralization of leukotoxin-induced pro-inflammatory response

**Figure 5:**
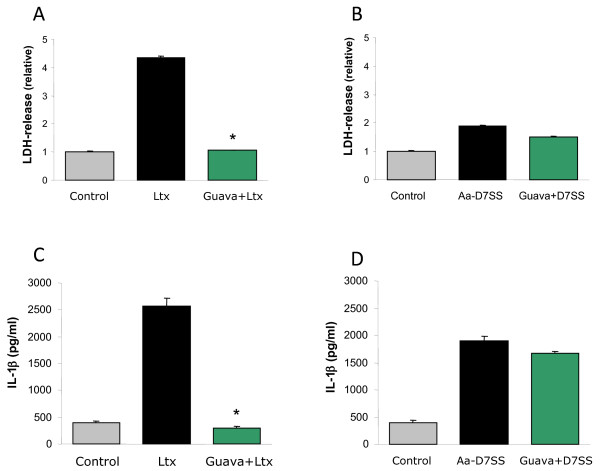
**Guava extracts neutralize leukotoxin-induced pro-inflammatory response, but not the bacteria-induced response.** LDH-release (**A **&**B**) and IL-1β secretion (**C** &**D**) of human leukocytes exposed to *A. actinomycetemcomitans* leukotoxin (100 ng/ml) (**A** &**C**) or bacteria from the leukotoxin mutant strain D7SSΔ*ltxA* (10 bacteria/leukocyte) (**B** &**D**) for 60 min. Mean ± SD of 3 experiments is shown and significant leukotoxin neutralization is indicated (*).

*A. actinomycetemcomitans* leukotoxin induces a rapid release of IL-1β from human leukocytes. The Guava extract (0.1%) efficiently inhibited (p ≤ 0.001) the release and activation of IL-1β that is induced in the presence of cytolytic concentrations of leukotoxin (100 ng/ml) (Figure 5A & C). On the contrary, the release of IL-1β caused by the leukotoxin mutant *A. actinomycetemcomitans* (D7SSΔ*ltxA*) that lacks leukotoxin was not interfered by the Guava extract (Figure 5B & D). This indicates a specific effect on the leukotoxin-induced leukocyte activation.

### Target identification of the Guava leukotoxin-neutralizing compounds

**Figure 6:**
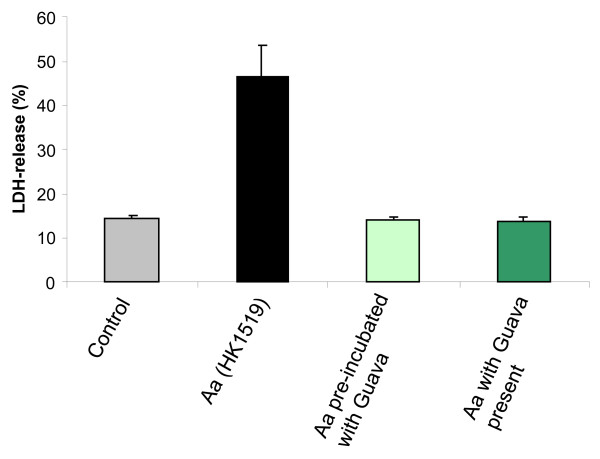
**The leukotoxin-neutralizing compounds bind to the bacteria-associated toxin upon neutralization.** LDH-release from macrophages (PMA-treated THP-1 cells) exposed to highly leukotoxic *A. actinomycetemcomitans* (strain HK1519) 10 bacteria/cell for 60 min. The macrophages were exposed to native bacteria, bacteria pre-treated with Guava extract (0.1%) or to bacteria in the presence of Guava (0.1%). Mean ± SD of 3 experiments is shown and significant leukotoxin neutralization is indicated (*).

To determine if the target molecules for the leukotoxin neutralization are on the bacterium or on the target cell, both leukotoxic bacteria and leukocytes were pre-exposed to Guava extracts. *A. actinomycetemcomitans* from the highly leukotoxic strain (HK1519) lost its leukotoxic activity (p ≤ 0.001) when pre-exposed to Guava extracts that were washed away before *A. actinomycetemcomitans* were added to the leukocytes (Figure 6). In addition, Guava extracts used for pre-exposure of leukotoxic bacteria lost their leukotoxin-neutralizing capacity. Leukocytes pre-exposed to Guava extract that was removed before addition of leukotoxin were still leukotoxin-sensitive. These results indicate that compounds in the Guava extract neutralize the leukotoxin by binding directly to the leukotoxin and not to the target leukocytes.

### Oral hygiene habits of Ghanaian adolescents

Among the 296 examined individuals, 295 used toothbrush and toothpaste daily for oral hygiene (Table 1). Furthermore, 56.4% of these adolescents also used chewing sticks from different plant materials for the same purpose. The most commonly used materials were Sokodua and Nim tree, which were used by 48% and 28.6%, respectively. Guava was used by 11.4% of the chewing stick users, which corresponded to 6.4% in the whole examined population.

**Table 1:** Usage of oral hygiene tools in a Ghanaian adolescent population

	Number/proportion
**Tooth brush/tooth paste**	
	Totally 295 (99.7%)
**Chewing sticks source**	
	Totally 167 (56.4%)
	Nim tree 50 (28.6%)
	Sokodua 84 (48.0%)
	Guava 20 (11.4%)
	Other 21 (12.0%)
**Cleaning frequency**	
	1 time/day 94 (31.8%)
	2 times/day 202 (62.2%)

Two hundred and ninety six (296) students at Kpandu Technical Institute, located in the Volta-region of Ghana, were examined.

## Discussion

The present study shows that an extract from Guava completely neutralizes the cytolytic and pro-inflammatory responses of human leukocytes exposed to *A. actinomycetemcomitans* leukotoxin. Compounds in the Guava extract bind to the toxin by a mechanism that is not yet known and completely abolishes its biological activity. Highly leukotoxic *A. actinomycetemcomitans* are frequently found in adolescents with periodontitis in some populations [23]. The highly leukotoxic JP2 clonal type of this bacterium has its origin from North- and West Africa, and a high prevalence of JP2 clone strains has been found in populations in this particular geographic area [13,16]. Data from the present study show that traditional chewing sticks are commonly used tools for oral hygiene practices in Ghana. More than 50% in the examined population reported daily use of chewing sticks obtained from different plants. The source of chewing stick material differs among subjects of various ethnicity and geographic origin [17]. The proportion of subjects who select Guava as source for chewing sticks was only 6.4% in the examined population, which is in line with previously presented data by Adu-Tutu and co-workers 1979 [17]. Our discovery that Guava releases components that neutralizes the activity of *A. actinmycetemcomitans* leukotoxin might be used in future preventive strategies in some populations. Several plants used as chewing stick material show antimicrobial activity against several bacterial species [20,21]. However, none of the plant extracts used in the present study inhibited growth of *A. actinomycetemcomitans*.

Guava (*Psidium guajava*) is grown in tropical and subtropical countries for food, but it is also widely used in folk medicine [24]. It is commonly used to treat gastrointestinal and respiratory disturbances and as an anti-inflammatory agent [25]. The ethanol extract of Guava leaves contains polyphenols, which have reported antioxidant, anti-cancer, anti-inflammatory and anti-allergic activities [26]. In addition, Guava extract contains compounds with anti-microbial activity, such as flavonoids, steroids, and tannins [27]. Since these compounds are commonly present in several plant species [28], the Guava-specific leukotoxin-neutralizing activity might be the result of a yet unknown compound. The molecular properties of the leukotoxin, such as the high iso-electric point (8.9), hydrophobic and hydrophilic regions, and the repeat region, mediate several binding targets for bioactive molecules [2]. This molecular structure of *A. actinomycetemcomitans* leukotoxin is shared by a number of proteins from the RTX family that are produced by a number of different Gram negative human pathogens [3]. This indicates that the bioactive leukotoxin-neutralizing components of Guava may also be used to inhibit the activity of RTX proteins from bacterial species other than *A. actinomycetemcomitans*.

Methods and tools for oral hygiene vary from country to country and from culture to culture. Despite the widespread use of toothbrushes and toothpastes, traditional methods of tooth cleaning using chewing sticks selected and prepared from the twigs, stems or roots from a variety of plant species have been practised for thousands of years in Asia, Africa, the Middle East, and the Americas [18]. Today, chewing sticks are used in many developing countries because of religion and/or tradition, and because of their availability, low cost, and simplicity [29]. In our recent investigation we found that >50% of the sample population in the Volta region of Ghana daily used chewing sticks for their oral hygiene. The consensus report on oral hygiene year 2000 stated that chewing sticks may have a role to play in the promotion of oral hygiene, and that evaluation of their effectiveness warrants further research [18].

The occurrence of the highly leukotoxic clone (JP2) of *A. actinomycetemcomitans* coincides geographically with the use of chewing sticks as tools for oral hygiene [23,29]. Our recent discovery of the unique property of Guava to release components that efficiently neutralize leukotoxin might be used in future strategies for periodontal treatment in these populations. This study shows for the first time that the selection of a particular plant for chewing stick might be of great importance in terms of having a therapeutic potential. The use of Guava as the source of the chewing stick material is not common in these populations [17], but was used by 5.6% of the examined Ghanaian population in the present study. However, it remains to be elucidated if neutralization of leukotoxin is an efficient treatment strategy for the aggressive form of peridontitis that is induced by the inflammatory response to the highly leukotoxic *A. actinomycetemcomitans.* Furthermore, a future aim will be to isolate the components in the Guava extract that are responsible for the leukotoxin neutralization. The clinical importance of the present discovery can be evaluated in parallel with the isolation of the active compounds. The history of Guava as source for medical treatments in traditional medicine indicates a low risk for negative side effects [25]. In addition, the results in the present study showed that Guava had no cytotoxic effects against the exposed human leukocytes. The Guava chewing stick can be the source of the leukotoxin-neutralizing compound, a well known and easily accessible tool for oral hygiene in many of the developing countries.

## Conclusions

The leukotoxin-neutralizing properties of Guava might be an important finding due to the high prevalence of highly leukotoxic *A. actinomycetemcomitans* in African countries where this plant material is easily available and used as an oral hygiene tool.

## Methods

### Bacterial cultures

In this study the following *A. actinomycetemcomitans* bacterial strains were used: *A. actinomycetemcomitans* strain HK 1519 (JP2 clone strain) and the D7SS strain with a complete leukotoxin promoter (non-JP2 clonal strain). In one experiment the leukotoxin knockout mutant strain D7SS Δ*ltxA* was used. The enhanced leukotoxin production of the JP2 clone is about 10 fold higher than the production of non-JP2 strains [9]. The *A. actinomycetemcomitans* strains were cultivated on blood agar plates at 37°C in aerobic atmosphere containing 5% CO_2_. After 72 h incubation the bacteria were harvested with a sterile cotton swab and suspended in 0.15 M NaCl. The optical density (OD^600^) was measured, and the bacteria were suspended in phosphate buffered saline (PBS) at different concentrations.

### Leukotoxin and leukocyte preparations

Leukotoxin was purified from *A. actinomycetemcomitans* strain HK 1519, described in details previously [30]. The purified leukotoxin was basically free from lipopolysaccharides (LPS) (<0.001% of total protein) and visualized by SDS-polyacrylamide gel separation.

Human leukocytes were isolated from an enriched leukocyte fraction (buffy coat) of venous blood (from blood donors at the University Hospital blood bank in Umeå, Sweden), as described previously [9]. Mononuclear leukocytes (MNLs) were isolated by isopycnic centrifugation in Lymphoprep® (Nycomed AB, Lidingö, Sweden). The fraction containing MNLs was collected, and the cells were washed 3 times with PBS (250 x *g*, 5 min) to remove platelets. The cell pellet was then re-suspended in culture medium RPMI-1640 containing L-glutamine, 10% foetal bovine serum (FBS), and Penicillin-Streptomycin (Sigma-Aldrich, St Louis, MI, USA) to yield 5 x 10^6^ cells/ml. In addition, in some experiments, Macrodex (Meda AB, Solna, Sweden) separation was used in order to isolate the leukocytes from the blood. This leukocyte fraction also contained neutrophils, and the procedure was performed as described earlier [9].

### Cell cultures

Cells of the human acute monocytic leukemia cell line THP-1 (ATCC 16) were cultured in RPMI-1640 (Sigma-Aldrich, St Louis, MI, USA) with 10% foetal bovine serum (FBS) (Sigma-Aldrich) at 37°C in 5% CO_2_. Before determination of leukotoxic activity, the THP-1 cells were seeded in 96-well cell-culture plates at a cell density of 5 x 10^5^ cells/ml in 100 μl culture medium supplemented with 50 nM phorbol 12-myristate 13-acetate (PMA, Sigma-Aldrich) and incubated for 72 h. The PMA-activated THP-1 cells exhibited adherent properties and enhanced sensitivity to the leukotoxin. Twenty-four h prior to leukotoxin exposure, the culture medium was discarded and 50 μl fresh medium without PMA was added to each well of the THP-1 monolayer

### Preparation of chewing stick extracts

Ethanol extracts from 7 different plant materials was performed by mixing minced plant materials with 70% ethanol (250 mg/ml). The mixtures were then gently agitated for 24 h before the insoluble material was removed by centrifugation (5 000 x g, 20 min), and the supernatants were frozen in aliquots at - 20°C before use. Twigs were studied from seven chewing stick sources: (1) Tropical almond (*Terminalia catappa)*, (2) Guava (*Psidium guajava*), (3) Blackberry (*Rhus*), (4) Nim tree (*Azadirachta*), (5) Sokodoa (*Garcinia*) family 1 and (6) family 2, and (7) chewing sponge (cellulose fibres of unspecified origin of spongy texture). Dry weight for the different extracts were in mg/ml; 1 = 8.9, 2 = 5.0, 3 = 2.5, 4 = 6.7, 5 = 5.9, 6 = 9.0 and 7 = 15.6. The twigs, including bark, were 4–8 mm in diameter and minced into pieces of about 1 x 1 mm before the extraction procedure. All extract revealed a strong yellow to green colour that was completely clear and without enhanced viscosity after the centrifugation. Leafs and fruits from Guava were also studied and extracted with the same procedure. The leaf extracts had a green colour and a dry weight of 10.0 mg/ml and the fruits revealed a light yellow colour in the extract with a dry weight of 3.5 mg/ml.

The 7 different chewing stick materials were initially collected from the Accra region of Ghana. A second collection of material solely from Guava was made in the Volta region of Ghana and contained twigs (including bark), leaves and fruits from this plant. This was made in order to check that the biological effect was associated to the Guava-plant and not to the growth conditions. In addition, the identification of the Guava plant was made by different individuals, which made the selection of material completely independent of each other in the first and the second collection procedure.

### Antimicrobial test

The different plant extracts were examined for possible inhibition of *A. actinomycetemcomitans* (strain HK1519 and D7SS) growth by 2 different methods.

*Inhibition of* A. actinomycetemcomitans *growth on agar*

One hundred μl of a bacterial suspension (≈2x10^9^ cells/ml) was spread on a blood agar plate with a sterile cotton swab, and then 10 μl of each extract was added. The plates were then cultured for 48 h at 37°C in 5% CO_2_ in air and the inhibition zones were examined in a microscope. Ten μl of 70% ethanol was used as a negative control.

*Inhibition of* A. actinomycetemcomitans *in broth*

One μl of a bacterial suspension (≈ 2 x 10^9^ cells/ml), 99 μl culture broth (peptone-yeast extract-glucose broth) [30] and 1 μl of the different stick extracts were put into 96-well culture plates. This yielded a final concentration of 1% extract in each well with broth cultured bacteria. The mixtures were then cultured for 48 h at 37°C in 5% CO_2_, and the optical density (OD) at 600 nm was documented automatically in a spectrophotometer. The increase in OD in each mixture was compared with that in a sample with 1% ethanol instead of a plant extract, and the % inhibition caused by the extracts was calculated.

### Cell survival and cell death analysis

The leukocyte preparations or THP-1 cells were mixed with the different extracts (≤0.1%) before leukotoxin (100 ng/ml) or leukotoxic bacteria (HK1519 10/cell) were added.

#### Cytolysis

Leukotoxin-induced cytolysis was determined by the release of the cytosol enzyme lactate dehydrogenase (LDH) as described earlier [30]. The cells were incubated for 60 min at 37°C in the presence of various concentrations of leukotoxin. The activity of the enzyme released from damaged cells into the supernatant was measured and expressed as a percentage of the total LDH activity released from cells lysed by exposure to 0.1% Triton X-100 for 60 min. In some experiments normalized values are shown with the spontaneous release from leukocytes in the control cultures set to 1.0. The different extracts alone had no effect on the activity of LDH when they were added to the positive control.

#### Propidium iodide (PI) uptake quantified by FACS (fluorescence assisted cell sorter)

The leukocytes were incubated in culture medium for 60 min at 37°C in the presence of 1 or 10 ng/ml *A. actinomycetemcomitans* leukotoxin. Leukocytes in plain culture medium served as controls. These cells were further analyzed for early detection of apoptosis/necrosis. The experiment was ended on ice and PI was added according to the Vybrant® Apoptosis Assay Kit #4 protocol (Molecular Probes/Invitrogen Labelling and Detection, Eugene, Oregon, USA). The cells were incubated for 30 min and transferred to FACS tubes and analyzed by FACS as soon as possible using 488 nm excitation with fluorescence emission at 575 nm (e.g., FL3) (Calibur, Becton Dickinson Immunocytometry Systems, CA, USA). Dead cells stained positive for PI, and the proportion was calculated from the results of 10 000 cells in each sample.

In addition, the different subsets of leukocytes (lymphocytes, monocytes and neutrophils) were identified through their specific pattern in forward- and side scatter of a FACS dot-plot. This gives an overview of which subsets of leukocytes were attacked by the leukotoxin and protected by chewing stick extract.

### Cytokine quantification

#### Enzyme-linked immunosorbent assay (ELISA)

The macrophages were exposed to bacterial stimuli as described above. The amounts of IL-1β secreted into the culture medium were determined by ELISA (Enzyme Linked Immuno Sorbent Assay) using DuoSet kits (R&D Systems Inc, Minneapolis, MN, USA). The experimental procedures were done according to the manufacturer’s protocol. This assay recognizes specifically the cleaved 17-kDa active form of IL-1β.

### Oral hygiene habits in Ghanaian adolescents

Two hundred ninety-six adolescents at the Kpandu Technical Institute in the Volta region of Ghana were interviewed about oral hygiene habits. They were 16–29 years of age, median age 19 years. The interview was made with a questionnaire and a brief clinical field examination of general oral health. Data from this survey was used only for to get an overview about chewing sticks usage and source of plant materials for the sticks in this population.

### Ethics

Blood was taken from donors visiting the University Hospital blood bank in Umeå, Sweden. Informed written approval was given by all subjects, and authorization for the study was granted by the Human Studies Ethical Committee of Umeå University, Sweden (§67/3, dnr 03–019). Ethical clearance for field studies in Ghana was approved by the University of Ghana (NMIMR-IRB CPN 049/08-09).

### Statistics

Student t-test was used to evaluate significant difference between tested samples and corresponding controls. P-values ≤0.05 were considered as significant differences between samples.

## Authors’ contributions

FK initiated the study, collected plant materials and supervised the field examination in Kpandu. DH performed the initial clinical trials and supervised the field examination in Kpandu. RG planned the extract methodology and advised in the plant biology topics. AJ conceptualized the study, conducted the analyses, and wrote the first draft of the manuscript. All contributed to and approved the final version of the submitted manuscript**.**

## Competing interests

The authors declare that they have no competing interests.
